# St. Luke's Hospital for the Insane

**Published:** 1889-12-28

**Authors:** 


### The Hospital Workshop.

### No. XIX.—ST. LUKE'S HOSPITAL FOR THE INSANE.

(By our Special Commissioner.)

This charity—the second of its name—stands in the heart of old St. Luke's parish, one of the most densely-populated districts in the whole of London. The present building cost £40,000, and was erected in 1780 by George Dunce, the same architect who designed Newgate. Traces of the gaol influence are visible in St. Luke's, which is large, massive, and—on the outside, at any rate—more or less suggestive of locks, bolts, and bars. A short while since the secretary, Mr. Percy de Bathe, kindly furnished your commissioner with much interesting information concerning the work of the hospital, both past and present.

The main entrance pierced an extremely thick wall that fronts the building and cuts it off from the busy thoroughfare. Some steps lead into what is called the "middle house," containing offices and residents' rooms. On each side of this central block are wings holding the wards, female on one side, male on the other, so that the plan of the hospital is sufficiently simple. Accommodation is hardly as large as external appearances would lead one to expect. This is partly due to the fact that the basement floor on the female side is disused because it is totally unfitted to meet the demands of modern treatment, while another on the male side is furnished with a stage and devoted to recreation purposes. After these deductions there is still space for 200 beds, of which 184 are occupied. In addition to this 190 patients are "out on trial"—that is to say, they have been sent home to their friends for a time to see how they get on away from the hospital.

On the first floor of the middle house is a landing, noteworthy as having been the scene of a little story that appeared long ago in *Household Words* under the title, "A Curious Dance Round a Curious Tree." This sketch was afterwards published as a pamphlet, and in that form is much prized by Dickens's collectors, a single copy lately fetching no less than £17 at a sale. Some people have questioned the exact authorship, but Mr. de Bathe states that he is in a position to prove that Charles Dickens wrote the article, and adds that his notes were taken from a corner of the landing already mentioned. The "curious tree" was a Christmas tree, around which the patients marched from the wards on either side, there being at that time no other place available for recreation. The walls are panelled with boards on which figure the names of former benefactors, with the amount of their donations. The letters are now nearly illegible, and the secretary states that to regild them would cost some hundreds of pounds. Their record proved useful in getting together the friends of the institution at its centenary, which was celebrated a few years since. Near this spot are the fire appliances, a department in which special care is taken, the attendants, for instance, being regularly drilled by a member of the Metropolitan Fire Brigade. The list of technical appliances looks very business like, and runs: "One copper branch. Five 50ft. lengths of 2in. canvas hose. One ⅝in. nozzle. Two hose and nozzle-spanners. Fire buckets and manual." Several rooms open upon this landing, the one at the back being used as a dining-room. It contains a quaintly shaped "Sheraton" sideboard, which may be taken as the type of many treasures of the kind scattered through the building. In front is the board-room, the walls of which are lined with paintings. One of the most striking is a full length portrait of the first president, Duke of Montague. The face is painted in a style that many critics maintain to be Gainsborough's, but the rest of the canvas appears to have been filled in by a very different hand. Another portrait is of Sir Thomas Clarke, dated 1754, a benefactor of the hospital to the extent of £30,000. A third is that of John Clark Powell, treasurer for the period of thirty-three years. The present holder of the post is a nephew, Mr. Arthur Powell, whose grandfather also filled the same office. Among other objects of interest are some "black jacks," huge leather vessels capable of holding one to two gallons apiece, and which were formerly used in the wards. There is also a fine old punch-bowl of transfer ware, ornamented with a plan of elevation of the hospital.

By the courtesy of Dr. Mickley, the medical superintendent, your commissioner was permitted to inspect the wards. The latter are warm and comfortable rooms, distinguished by letters, and one of them may be briefly described. Let the reader picture to himself a long red-carpeted corridor, some sixteen feet across, lighted with numerous windows down one side, and having a row of sleeping-rooms on the other. The centre widens out into a space that stretches across the whole breadth of the wing. There are several large stove-fireplaces, carefully protected by wire guards. Each ward contains a piano, there are bagatelle boards, and other amusements, and a plentiful supply of books. Birds, plants, and flowers serve to enliven the scene. Indeed, one could hardly imagine a greater contrast than that between the prison-like exterior of the hospital and the comfort that reigns inside. Some of the wards have dados of oak-grained wainscoting, which is also used in the bedrooms. At the moment of entering the first female ward, a talkative patient was detailing her woes at a great rate. Having laid down the general principle that none had any right to tell lies to the committee she proceeded to inform us that one of the officials was not a gentleman by birth, but that in point of fact his mother was a charwoman with whom she, the speaker, had been intimately acquainted. These statements were followed up by a good many acrid reflections on the personal character of the individual in question, and finished up with a strong assurance that we, her visitors, the committee and the whole wide world should hear about these things whether we liked it or not. Most of the other patients in the ward were sitting quietly about, sewing, reading, and, in a few instances, dozing or staring into vacancy, with the unmistakable apathy of melancholia. They appear to be drawn from a fairly good class. Pauper cases are not admitted, neither are epileptics, nor those suffering from general paralysis. Our inspection revealed the usual phases of mental disease, such as melancholia, maniacal, delusional, and other forms of insanity. One of the women was making extremely pretty penwipers. To use her own words, she had "cut out the little black boy from Pears' soap, don't you know?" This figure, provided with a red satin cap and a blue dress, she fixed on a piece of black cloth. The result was a most artistic penwiper, which quite justified the verdict of another patient, "Well, I think it very clever."

(*To be continued.*)

